# Draft Genome Sequence of *Mycobacterium lentiflavum* CSUR P1491

**DOI:** 10.1128/genomeA.00817-15

**Published:** 2015-07-23

**Authors:** Michael Phelippeau, Olivier Croce, Catherine Robert, Didier Raoult, Michel Drancourt

**Affiliations:** Aix Marseille Université, URMITE, UMR CNRS 7278, IRD 198, INSERM 1095, Faculté de Médecine, Marseille, France

## Abstract

We announce the draft genome sequence of *Mycobacterium lentiflavum* strain CSUR P1491, a nontuberculous mycobacterium responsible for opportunistic potentially life-threatening infections in immunocompromised patients. The genome described here comprises a 6,818,507-bp chromosome exhibiting a 65.75% G+C content, 6,354 protein-coding genes, and 75 RNA genes.

## GENOME ANNOUNCEMENT

The initial description of *Mycobacterium lentiflavum* was based on three isolates belonging to a single DNA homology group (1). Further phylogenetic analyses found >99.5% similarity in the 16S rRNA gene sequence with *Mycobacterium simiae* and *Mycobacterium genavense* (1). This observation qualified *M. lentiflavum* as a member of the *Mycobacterium simiae* complex. This environmental organism (2) has been isolated from human specimens, including an intervertebral disc (1), respiratory tract samples and gastric/gut aspirates (1, 3), lymph nodes (4), feces (5), and bone marrow (5). *M. lentiflavum* emerges in cystic fibrosis patients (6) and was responsible for the death of one heart-transplanted immunocompromised patient (5). Routine identification of this fastidious mycobacterium can be achieved by partial *rpoB* gene sequencing (7).

We sequenced the whole genome of *M. lentiflavum* to exact the phylogenetic relationships within the *M. simiae* complex, design tools for its genotyping, and disclose any genotypic pattern possibly related to host-mycobacterium relationships.

Genomic DNA was extracted from *M. lentiflavum* strain CSUR P1491 (isolated from the sputum of a cystic fibrosis patient) grown on Middlebrook 7H10 solid medium at 37°C in a 5% CO_2_ atmosphere for 3 weeks. Genomic DNA was sequenced on Illumina MiSeq throughout three runs, including one paired-end Nextera and two mate pair libraries, in a 2 × 250-bp run for each barcoded library. The whole set of reads was trimmed using Trimmomatic (8), then assembled through the assembler software SPAdes v3.5 (9, 10). Contigs obtained were combined together by SSPACE v2 (11), Opera v2 (12) helped by GapFiller v1.10 (13), and homemade tools to refine the set.

The draft genome of *M. lentiflavum* consists of five contigs without gaps, containing 6,818,507 bp. The G+C content of this genome is 65.75%. Noncoding genes and miscellaneous features were predicted using RNAmmer (14), ARAGORN (15), Rfam (16), Pfam (17), and Infernal (18). Coding DNA sequences (CDSs) were predicted using Prodigal (19) and functional annotation was achieved using BLAST+ (20) and HMMER3 (21) against the UniProtKB database (22). This genome is predicted to encode at least 75 predicted RNAs, including 3 rRNAs, 52 tRNAs, 1 transfer-messenger RNA (tmRNA), and 19 miscellaneous RNAs. A total of 6,354 genes were identified, representing a coding capacity of 6,173,541 bp (coding percentage, 90.54%). Whereas 4,369 genes matched a least one sequence in the Clusters of Orthologous Groups (COGs) database (23, 24, 25) with BLASTP default parameters, 1,150 (18.1%) were assigned as hypothetical proteins, and 332 (5.23%) genes were founded as encoding putative protein. No genes encoding antibiotic resistance were found, but bleomycin resistance is encoded.

*In silico* DNA-DNA hybridization (DDH) was performed with the genome-to-genome distances between pairs of two closed genomes (25). The genome of *M. lentiflavum* was locally aligned 2-by-2 using the BLAT algorithm (26, 27) against *M. simiae* and *M. genavense*. The DDH was estimated from a generalized linear model using a specific distance formula (28), resulting in following values: 27.5% (±2.43) with *M. simiae* and 34.6% (±2.47) with *M. genavense*. These data confirm *M. lentiflavum* as a unique species clearly distinct from *M. simiae* and *M. genavense*.

### Nucleotide sequence accession numbers.

The *M. lentiflavum* strain CSUR P1491 genome sequence has been deposited at EMBL under the accession numbers CTEE01000001 to CTEE01000005.
